# Standards-Based Clinical Decision Support Platform to Manage Patients Who Meet Guideline-Based Criteria for Genetic Evaluation of Familial Cancer

**DOI:** 10.1200/CCI.19.00120

**Published:** 2020-01-17

**Authors:** Guilherme Del Fiol, Wendy Kohlmann, Richard L. Bradshaw, Charlene R. Weir, Michael Flynn, Rachel Hess, Joshua D. Schiffman, Claude Nanjo, Kensaku Kawamoto

**Affiliations:** ^1^ Department of Biomedical Informatics, University of Utah, Salt Lake City, UT; ^2^ Huntsman Cancer Institute, University of Utah, Salt Lake City, UT; ^3^ Department of Internal Medicine, University of Utah, Salt Lake City, UT; ^4^ Department of Population Health Sciences, University of Utah, Salt Lake City, UT; ^5^ Department of Pediatrics, University of Utah, Salt Lake City, UT

## Abstract

**PURPOSE:**

The ubiquitous adoption of electronic health records (EHRs) with family health history (FHH) data provides opportunities for tailoring cancer screening strategies to individuals. We aimed to enable a standards-based clinical decision support (CDS) platform for identifying and managing patients who meet guidelines for genetic evaluation of hereditary cancer.

**METHODS:**

The CDS platform (www.opencds.org) was used to implement algorithms based on the 2018 National Comprehensive Cancer Network guidelines for genetic evaluation of hereditary breast/ovarian and colorectal cancer. The platform was designed to be interfaced with different EHR systems via the Health Level Seven International Fast Healthcare Interoperability Resources standard. The platform was integrated with the Epic EHR and evaluated in a pilot study at an academic health care system.

**RESULTS:**

The CDS platform was executed against a target population of 143,012 patients; 5,245 (3.7%) met criteria for genetic evaluation based on the FHH recorded in the EHR. In a clinical pilot study, genetic counselors attempted to reach out to 71 of the patients. Of those patients, 25 (35%) scheduled an appointment, 10 (14%) declined, 2 (3%) did not need genetic counseling, 7 (10%) said they would consider it in the future, and 27 (38%) were unreachable. To date, 13 (52%) of the scheduled patients completed visits, and 2 (15%) of those were found to have pathogenic variants in cancer predisposition genes.

**CONCLUSION:**

A standards-based CDS platform integrated with EHR systems is a promising population-based approach to identify patients who are appropriate candidates for genetic evaluation of hereditary cancers.

## INTRODUCTION

Increasing evidence supports individualizing cancer screening based on risk, with a selective application of specific screening technologies best suited to the individual.^1-8^ Individuals at higher risk for cancer may benefit from more aggressive screening and risk reduction strategies. Family health history (FHH) is one of the most valuable pieces of information for estimating cancer risk, including breast and colorectal cancer.^9,10^ FHH-based estimates indicate that the prevalence of individuals with a new diagnosis of cancer and familial risk is 12% for breast cancer and 5% for colorectal cancer.^11^ Genetic testing is an important tool for further specifying risks for people with a concerning family history, and guidelines for managing cancer risk associated with specific hereditary cancer predispositions are also being developed.^12,13^

Effective interventions are needed to identify patients who meet the criteria for genetic testing for hereditary cancer. A promising solution is to automate the identification and management of patients who meet criteria for genetic evaluation using electronic health record (EHR) technologies coupled with clinical decision support (CDS) tools. However, implementation of FHH collection, FHH assessment, and referral to genetic services is quite challenging in today’s overwhelmed primary care environment, even in the presence of CDS.^14,15^ According to a systematic review of CDS interventions,^16^ previous approaches to managing cancer risk consisted primarily of nontailored CDS reminders to providers and patients when routine cancer screening was due.^17-21^ Two studies investigated CDS interventions focused on improving guideline-based referrals to genetic counseling for patients with familial risk for breast and colorectal cancer.^22,23^ In both studies, primary care providers used the CDS to create a pedigree, evaluate risk, and refer eligible patients to genetic counseling services.

**Context**

**Key Objective**
To enable and pilot a standards-based clinical decision support (CDS) platform for identifying and managing patients who meet guidelines for genetic evaluation of hereditary cancer based on family health history in the electronic health record.
**Knowledge Generated**
The CDS platform was successfully piloted at the University of Utah Health and the Huntsman Cancer Institute. The platform identified a relatively large number of patients who could benefit from genetic evaluation, including individuals who were later found to have pathogenic variants in cancer predisposition genes.
**Relevance**
The proposed approach could serve as a national model for population-based identification, outreach, and tracking of patients who may benefit from genetic evaluation of risk for familial cancer and personalized cancer screening.

CDS is considered to be a critical component for optimally applying the latest research findings to patient care.^24^ With the meaningful use incentive program, EHR adoption in US hospitals has increased to > 95%.^25^ This environment provides a unique opportunity for delivering effective CDS to patients and providers on a large scale. Yet, despite supporting evidence and federal incentives for CDS adoption, the use of CDS for tailored cancer screening is still limited.^16^ Critical barriers include EHR systems with limited CDS capabilities,^26^ the high cost of CDS logic development, and minimal sharing of CDS among health care organizations.^27^ EHR-agnostic and standards-based CDS platforms are a promising approach to enable sharing of advanced CDS capabilities across health care organizations.^28^

The overall goal of this project was to investigate a CDS platform for identifying appropriate candidates for hereditary cancer genetic testing designed to be used at multiple organizations using different EHRs. Unlike previous studies, our CDS platform uses a population health management (PHM) approach that leverages FHH information already available in the EHR to enable genetic counseling teams to identify and manage patients who meet guideline-based criteria for genetic evaluation of hereditary cancer. We describe the (1) CDS clinical workflow integrating primary care and genetic counseling settings; (2) CDS logic based on National Comprehensive Cancer Network (NCCN) criteria for breast and colorectal cancers^12,13^; (3) software architecture leveraging the OpenCDS platform^29,30^; and (4) results of a clinical pilot study at University of Utah Health (UHealth) and the Huntsman Cancer Institute.

## METHODS

### CDS Workflow

To guide the design of the CDS intervention, we conducted a mixed-methods workflow analysis to determine how FHH is documented and used for clinical decision making in primary care. The study was approved by the University of Utah institutional review board. In prior work, we surveyed and interviewed 96 medical assistants and 40 providers at 10 primary care clinics at UHealth.^31^ Briefly, the following insights derived from the FHH analysis guided the design of the PHM CDS workflow:

1. Fragmented documentation and inconsistent use of FHH in primary care create gaps that may prevent eligible patients from being offered genetic evaluation. Prior to the CDS platform, there was no systematic process in place to identify primary care patients who met FHH-based criteria for genetic evaluation of hereditary cancers.
2. Primary care providers are overwhelmed with other clinical priorities that prevent systematic documentation and use of FHH in patient care. Alternative approaches should minimize additional effort from primary care providers, while still keeping them informed through a common mechanism.
3. Although FHH collection at primary clinics is far from perfect, the clinics often collect sufficient information to evaluate FHH-based criteria for hereditary cancer evaluation, possibly with low sensitivity but high specificity.
4. Frequent use of the structured FHH section as a part of visit intake by medical assistants allows a simpler implementation approach that could be later enhanced with more sophisticated natural language processing (NLP) methods to extract FHH from visit notes.

### CDS Logic

We developed rule-based algorithms to identify individuals who meet guideline-based criteria for genetic evaluation of risk for familial breast and colorectal cancer. The algorithms were adapted from the NCCN guidelines for genetic evaluation of familial cancers.^12,13^

### Software Architecture

The CDS platform uses a combination of open-source CDS software (OpenCDS: www.opencds.org) integrated with tools available in commercial EHR systems, including a patient portal and a PHM platform. To maximize interoperability of the CDS platform with other health care organizations and EHR systems, we adopted a set of standard application program interfaces (APIs) that are increasingly gaining adoption by EHR systems, notably the Health Level Seven International (HL7) Fast Healthcare Interoperability Resources (FHIR)^32^ and CDS Hooks APIs. The first implementation was integrated with the Epic EHR at UHealth. Integration with Epic at New York University (NYU) and Cerner at Intermountain Healthcare are underway.

### Clinical Pilot Study at UHealth and Huntsman Cancer Institute

We conducted a pilot observational study of the CDS platform with patients assigned to 5 UHealth primary care providers. We collected descriptive statistics, including the number of patients who were screened by the algorithm; met criteria for genetic evaluation; met criteria and had not had genetic counseling previously; received and responded to outreach communication via the patient portal; received and responded to outreach communication via a telephone call; agreed to schedule an appointment for genetic counseling, did not agree to schedule an appointment and could not be contacted; completed a genetic counseling appointment; received genetic testing; and had a positive genetic test for a pathogenic variant of a gene implicated in cancer predisposition.

## RESULTS

### CDS Workflow

**FIG 1.**
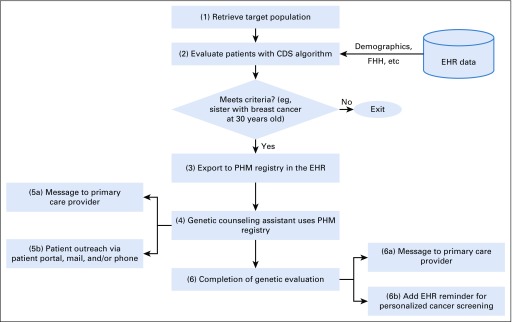
Clinical decision support (CDS) workflow. The numbers in each rectangle correspond to the workflow steps described in the text. EHR, electronic health record; FHH, family health record; PHM, population health management.

The resulting CDS workflow consists of the following steps (Fig 1):

Step 1. A computer algorithm retrieves a target population for evaluation based on data such as demographics and last activity with the health care system.
Step 2. Each patient is evaluated against the algorithms adapted from NCCN guidelines for genetic evaluation of breast and colorectal cancer (see CDS Logic section).
Step 3. Patients who meet the criteria are exported to a registry in the EHR’s PHM platform (Healthy Planet in Epic).
Step 4. Genetic counseling assistants working under the supervision of a genetic counselor use the PHM platform to review patients’ information, send and track responses to patient outreach communication, and track the status of each patient in the genetic evaluation workflow.
Step 5. (a) One week prior to sending an outreach message to the patient, a message is sent to the designated primary care provider notifying him or her that the patient meets the criteria and will be contacted. (b) Next, an outreach message is sent via the patient portal or by mail, depending on the patient preference noted in the EHR. Three attempts are made by phone to contact patients who have not responded to the original outreach message.
Step 6. (a) Outcomes of the genetic evaluations are documented in the EHR. Patients and primary care providers receive a summary letter, including a personalized risk assessment based on FHH and genetic testing, along with risk-appropriate screening recommendations. (b) As a future enhancement, if the evaluation warrants changes to the patient’s cancer screening plan, the genetic counselor will add or modify (eg, increases in frequency, changes in imaging modality) an EHR reminder that notifies the patient and the primary care provider when the patient is due for screening.

### CDS Logic

The CDS algorithm consists of criteria for the target population for risk evaluation and criteria for genetic counseling referral based on the FHH in the EHR. At UHealth, the target population for risk evaluation consisted of 25- to 60-year-old patients who had at least 1 primary care visit at UHealth in the last 3 years. The criteria for genetic evaluation are provided in Table 1.

**TABLE 1.**
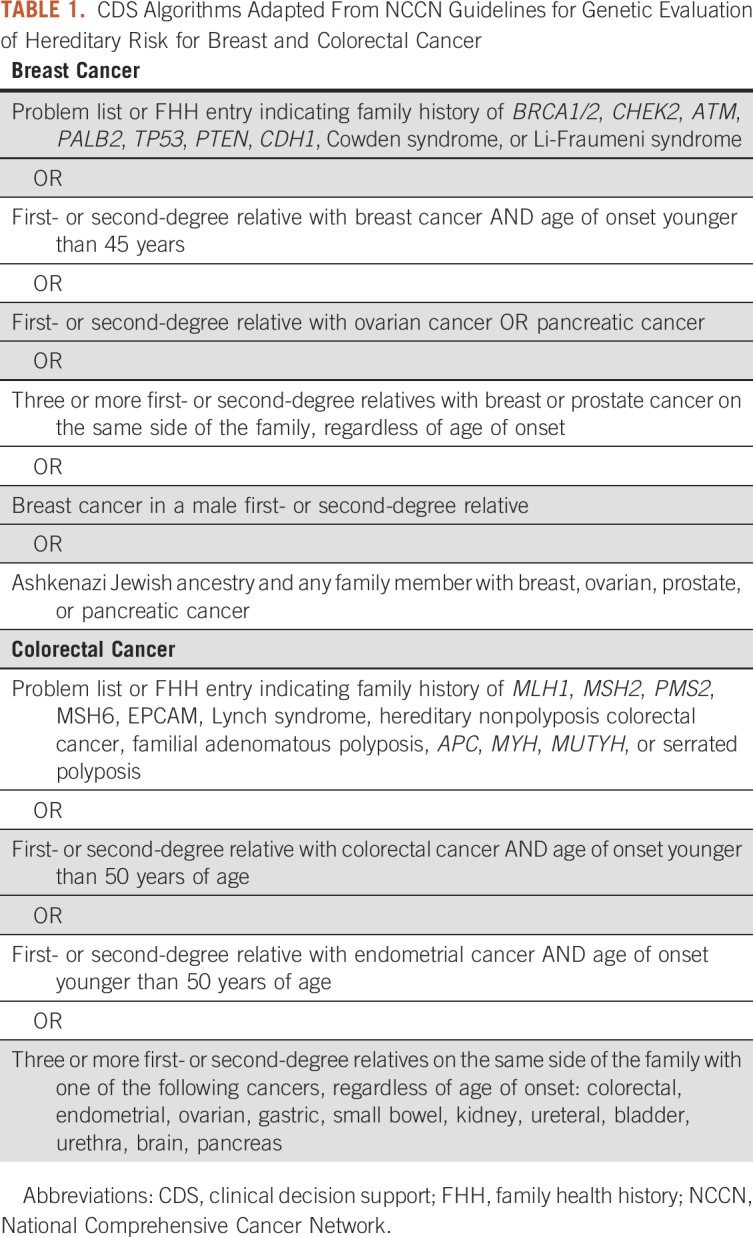
CDS Algorithms Adapted From NCCN Guidelines for Genetic Evaluation of Hereditary Risk for Breast and Colorectal Cancer

### Software Architecture

**FIG 2.**
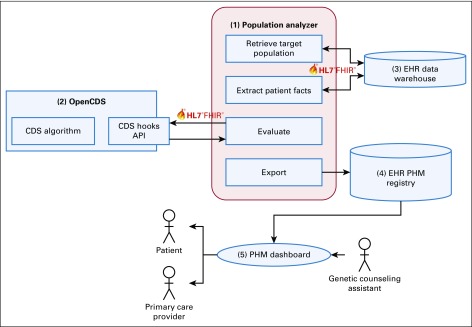
Clinical decision support (CDS) software architecture. Numbers correspond to component descriptions in the text. API, application program interfaces; EHR, electronic health record; HL7, Health Level Seven International; FHIR, Fast Healthcare Interoperability Resources; PHM, population health management.

The CDS platform includes the following components (Fig 2). (1) The Population Analyzer retrieves the target population for screening, extracts patient facts from the EHR, communicates with OpenCDS for algorithm execution, and exports patients who meet criteria to the PHM registry. The patient facts include age, sex, clinical encounters (type, date, status, and location), patient religion, and FHH (date captured, relative diagnosis, relative type, age of onset, and optional textual comments). (2) OpenCDS executes algorithms to determine patient eligibility for genetic evaluation of hereditary cancers. (3) The EHR’s data warehouse provides patient data for CDS evaluation. (4) The EHR's PHM registry stores patients who meet criteria for genetic evaluation. (5) The EHR’s PHM platform provides patient outreach and tracking, and provider communication functions.

The Population Analyzer and OpenCDS are open-source software, whereas EHR components are proprietary and vendor specific. The Population Analyzer communicates with EHR components through interoperable APIs compliant with the HL7 FHIR standard. Also, the Population Analyzer communicates with OpenCDS through a Web services API compliant with the HL7 CDS Hooks standard. The CDS platform has been deployed in a production environment at UHealth, and deployment at NYU is underway.

### Clinical Pilot Study at UHealth and Huntsman Cancer Institute

**FIG 3.**
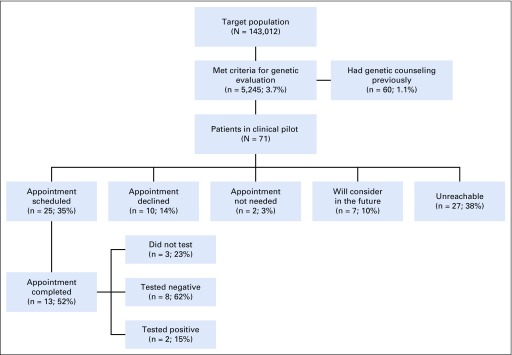
Results of clinical pilot study conducted at University of Utah Health and Huntsman Cancer Institute.

The pilot was carried out from September 12, 2018, to July 5, 2019. The CDS platform was executed with a target population of 143,012 patients (Fig 3); 5,245 patients (3.7%) met criteria for genetic evaluation (4,810 breast, 562 colorectal, and 127 both). Of 5,245 patients, only 60 (1.1%) had received genetic counseling at the Huntsman Cancer Institute that was documented in the EHR before the CDS platform was implemented. Those individuals were excluded from outreach for genetic counseling. Of the remaining 5,185 patients, a genetic counseling assistant attempted to reach out to a sample of 71 patients seen by 1 of the 5 primary care providers who participated in the pilot. Of those 71 patients, 25 (35%) scheduled an appointment, 10 (14%) were not interested, 7 (10%) said they would consider it in the future, 27 (38%) were unreachable, and 2 (3%) were considered to not need an appointment. Three patients (4%) reported prior genetic counseling themselves, which was not documented in our EHR, or counseling for an affected family member. One patient still had an appointment. Of the 13 patients who completed a genetic counseling visit as of October 2019, 3 (23%) did not undergo testing, 8 (62%) tested negative, and 2 (15%) tested positive. One patient had FHH documentation of cancer in a nonblood relative in the EHR. Of 13 patients who had their FHH reviewed by a genetic counselor, 10 (77%) had additional FHH that had not been documented in the EHR.

### Dissemination Efforts

The CDS platform was also implemented with the Epic EHR at NYU in preparation for a multisite randomized controlled trial. Of 311,957 patients at NYU, 16,850 (5.4%) met criteria for genetic evaluation of breast (15,701) and/or colorectal (1,640) cancer. We have also initiated efforts with Intermountain Healthcare to test the interoperability of the CDS platform with the Cerner EHR.

## DISCUSSION

We have implemented a CDS platform that enables PHM of individuals who meet guideline-based criteria for genetic evaluation of risk for familial cancers based on FHH documentation in the EHR. The approach has several strengths, including building on current primary care provider workflows and workload without creating any additional burden; flexibility to account for variation in FHH documentation accuracy and workflows; use of relatively simple, guideline-based evaluation criteria that do not require a complete FHH pedigree; and use of open-source software that interfaces with vendor-based EHR systems via standard-compliant APIs. Limitations include reliance on EHR FHH documentation that is often inaccurate or incomplete^33^; inability to accurately distinguish patients who have left the health care system; labor-intensive patient outreach and coordination of appointments; and need for increased access to genetic counselors to meet the demand generated by increased identification. The proposed approach has the potential to be implemented at multiple health care organizations using different EHRs, serving as a national model for identifying patients who may benefit the most from genetic evaluation of risk for familial cancers and personalized cancer screening.

In the clinical pilot study, 35% of the sampled patients scheduled an evaluation with a genetic counselor. It is possible that the reach rate would be higher if patients were directly referred by their primary care provider. However, the 2 previous trials investigating primary care–driven approaches to familial cancer risk management reported adoption challenges. The first had low provider adoption with no improvements in referral rates.^23^ The second had a 2-fold increase in genetic counseling referrals, but the intervention required training of a clinician at each site to be in charge of all visits involving potential referrals, and familial cancer risk visits took a median of 28 minutes.^22^ Future studies could investigate a hybrid approach where patients are first identified and contacted through a population-based approach. For those who are unreachable, a reminder could be placed in the EHR for the primary care provider to discuss at the next patient appointment.

A multisite trial is underway at UHealth and NYU to investigate ways to maximize the efficiency of providing genetic counseling and testing, including a self-directed approach that leverages chatbot technology for pretest counseling and return of negative genetic test results. Extrapolated to the entire target populations at UHealth and NYU, the CDS platform may benefit approximately 21,000 patients who meet criteria for genetic evaluation at the 2 institutions.

The CDS platform software was designed to interface with different EHR systems through standard interfaces, and the proposed workflow was designed to support variation in FHH documentation practices with minimal impact on primary care providers. These design features were deliberately incorporated to support implementation at multiple health care organizations. Nevertheless, we anticipate several technical challenges in disseminating the proposed approach. First, CDS Hooks adoption among EHR vendors is still low; FHH does not have a published FHIR profile supported by EHR vendors,^34^ and standards-based population data retrieval methods are still under development at HL7. For the UHealth and NYU implementations, we developed custom adaptations to retrieve and translate data from Epic to FHIR at the population level. At this point, similar data adaptors would need to be developed for other EHR products until FHIR adoption becomes more standardized and universal, including for accessing FHIR data for large populations in bulk. Second, because most EHR vendors support customization, even organizations using the same EHR product may create and use different terms to describe diseases and family relationships in FHH documentation. To address this challenge, we developed a term-mapping framework to support the creation of mappings between local terms and standard vocabularies. Third, local variation in information technology infrastructure (eg, network configurations, server access policies) at UHealth and NYU required some local customization in the Population Analyzer for population retrieval and FHH extraction.

The CDS platform does not require collection of a complete FHH pedigree, which would impose important changes to primary care workflow. According to our workflow analysis, despite substantial variation within and among providers and clinics, FHH is generally documented as structured data in the FHH section, most often by medical assistants during clinic intake. Yet, structured FHH data are often supplemented with short free-text comments about the relationship (eg, “aunt” as structured data and “maternal side” in the comments) and age of onset to express fuzzy ranges and uncertainty (eg, early 30s, premenopausal, early onset). To address this challenge, we have developed NLP algorithms that extract information from short comment fields in the FHH section.^35^ The algorithm achieved 98% sensitivity and 100% precision for the extraction of age of onset, increasing the number of FHH entries with computable age of onset by 50%. Integration of the NLP pipeline with the CDS platform is underway.

This study has several limitations. First, the CDS platform relies on EHR FHH documentation that is often incomplete.^33^ To address this challenge, the CDS platform aims for a high positive predictive value rather than high sensitivity. Of the 71 patients in the pilot, only 1 was determined to be false positive. Second, the pilot study was conducted at a single health system, using tools within 1 commercial EHR and based on FHH documentation workflows (eg, medical assistants collecting FHH as a part of a visit intake) that may be different from those used elsewhere. At the same time, our screening algorithms detected a similar rate of patients at NYU who meet criteria for genetic evaluation, suggesting that generalizability to other institutions may be possible. Third, we are unable to distinguish patients who have left the health care system and may be less likely to respond to outreach attempts. Last, although the CDS platform automates the screening of patients who meet NCCN-based criteria, it still relies on labor-intensive patient outreach, coordination of appointments, and genetic counseling visits.

In conclusion, a standards-based CDS platform was successfully integrated with a currently existing EHR at 2 academic medical centers (UHealth and NYU) to identify, reach out to, and provide genetic counseling to patients who met criteria for genetic evaluation of risk for familial cancers. In a pilot study, genetic counselors reached out to 71 patients identified by the platform. Of those tested, 15% harbored a pathogenic variant in a cancer predisposition gene. A randomized controlled trial comparing different approaches for providing genetic counseling and testing is underway at UHealth and NYU. The proposed approach could serve as a national model for population-based identification, outreach, and tracking of patients who may benefit from genetic evaluation of risk for familial cancer and personalized cancer screening.
